# The feline bile salt export pump: a structural and functional comparison with canine and human Bsep/BSEP

**DOI:** 10.1186/1746-6148-9-259

**Published:** 2013-12-20

**Authors:** Cyrina D van Beusekom, Jeroen JMW van den Heuvel, Jan B Koenderink, Johannes A Schrickx, Frans GM Russel

**Affiliations:** 1 Veterinary Pharmacology, Pharmacotherapy and Toxicology, Institute for Risk Assessment Sciences, Faculty of Veterinary Medicine, Utrecht University, Yalelaan 104, Utrecht 3584 CM, The Netherlands; 2 Department of Pharmacology and Toxicology, Radboud University Nijmegen Medical Centre, Nijmegen Centre for Molecular Life Sciences, Geert Grooteplein 28, Nijmegen 6500 HB, The Netherlands

**Keywords:** BSEP, ABCB11, Transporter, Cat, Dog, Liver, Drugs, Toxicity, Bile acids, Inhibitor

## Abstract

**Background:**

The bile salt export pump (BSEP/ABCB11) is the primary transporter for the excretion of bile acids from hepatocytes into bile. In human, inhibition of BSEP by drugs has been related to drug-induced cholestasis and subsequent cytotoxic effects. The role of BSEP in canine and feline liver diseases has not been studied in detail, but the same mechanism of inhibition by drugs as in humans could play a role in veterinary medicine. The aim of this study was to investigate the functional characteristics of feline Bsep in comparison with canine and human Bsep/BSEP with respect to substrate affinities and inhibitory potential of model drugs. Orthologs of all three species were cloned and cell membrane vesicles overexpressing feline, canine and human Bsep/BSEP were prepared for functional analyses.

**Results:**

The cDNA sequences of the open reading frames of feline, canine and human Bsep/BSEP showed a high similarity between the species. Functional studies demonstrated for all species a tendency to a higher affinity of BSEP/Bsep for the conjugated bile acid taurocholic acid (TCA) than glycocholic acid (GCA), and a higher affinity for GCA than for the unconjugated cholic acid (CA). The inhibitory potency of the model inhibitors cyclosporine A, troglitazone and ketoconazole was characterized against TCA uptake into BSEP/Bsep containing membrane vesicles. All three substances potently inhibited TCA uptake without significant species differences.

**Conclusion:**

Structure and functional characteristics of cat, dog and human Bsep/BSEP appeared to be very similar, indicating that the properties of this transporter have been highly preserved among the different species. Therefore, inhibition of BSEP by drugs could also be a mechanism in cholestasis and liver disease in veterinary relevant animal species. This model could be used to predict drug-induced liver injury caused by BSEP inhibition at an early stage in veterinary drug development.

## Background

An important function of the liver is the formation of bile, which is composed of bile salts, phospholipids and organic anions. Bile salts are amphipathic molecules that have detergent properties and can be highly toxic to hepatocytes if they accumulate intracellularly. Membrane-bound transporter proteins are essential in the transport of bile by the liver. The ATP-binding cassette (ABC) transporter, bile salt export pump (BSEP/ABCB11), is located in the canalicular membrane of the hepatocyte where it actively secretes bile salts into the bile at the expense of ATP hydrolysis.

In human medicine, inhibition of ABC transporters by drugs has been implicated in various adverse drug reactions of which drug-induced liver injury has been related to inhibition of BSEP in the liver. The inhibition of bile acid secretion leads to high intracellular bile acid concentrations and subsequent cytotoxic effects [1]. The clinical relevance of BSEP in bile salt secretion in man has also been demonstrated by several genetic traits, such as progressive familial intrahepatic cholestasis type 2 (PFIC2), benign recurrent intrahepatic cholestasis (BRIC), and intrahepatic cholestasis of pregnancy (ICP) (for review see [2,3]).

One of the first drugs that turned out to be a BSEP inhibitor was troglitazone and it was withdrawn from the market because of the development of cholestatic liver injury [4]. To predict drug-induced liver injury caused by BSEP inhibition at an early stage in drug development, *in vitro* assays have been developed using membrane vesicles from genetically engineered cells overexpressing human or rat BSEP/Bsep [5]. Several known cholestatic drugs showed BSEP inhibition in these membrane vesicles, including cyclosporine A, rifampicin and cloxacillin, suggesting that BSEP inhibition is the mechanism behind their hepatotoxic potential [6].

The causes and prevalence of liver diseases in dogs and cats are mostly unknown [7], but have also been related to drugs [8,9]. The role of BSEP in canine and feline liver diseases has not been studied in detail, but the same mechanism of inhibition by drugs as in humans could play a role in veterinary medicine. Recently, inhibition of the bile salt export pump and multi-drug resistance-associated protein (mrp) 2 by a novel kinase inhibitor was found to be related to the development of severe hepatotoxicity in dogs [10]. Previously, the canine Bsep has been cloned and partly functionally characterized to aid the extrapolation of toxicological data from dogs to humans [11]. However, data on feline Bsep is completely absent.

The aim of this study was to investigate the functional characteristics of feline Bsep in comparison with canine and human Bsep/BSEP with respect to substrate affinities and inhibitory potential of model drugs. Knowledge about feline Bsep is lacking and therefore, this is the first study in which the feline Bsep has been cloned and characterized. As a model for *in vitro* cross-species extrapolation of hepatotoxic data, we cloned BSEP/Bsep of all three species and prepared cell membrane vesicles for functional analyses.

## Methods

### Chemicals and reagents

Tauro [carbonyl-^3^H]cholic acid (TCA) (5 Ci/mmol) was obtained from Perkin Elmer (Boston, MA). Cholic acid [2,4-^3^H] (CA) (30 Ci/mmol) and glycocholic acid[glycine-2-^3^H] (GCA) (40 Ci/mmol) were purchased from Biotrend (Köln, Germany). Adenosine triphosphate (ATP), adenosine monophosphate (AMP), cholic acid, cyclosporine A, glycocholic acid, ketoconazole, taurocholic acid, troglitazone were purchased from Sigma Aldrich (St. Louis, MO, USA). Bac-to-Bac and Gateway systems, Dulbecco’s modified Eagle’s medium-GlutaMAX-I culture medium and fetal calf serum were obtained from Invitrogen (CA, USA). Triple flasks (500 cm^2^) were purchased from Sanbio BV Biological Products (Uden, The Netherlands).

### RNA isolation and cDNA synthesis

Liver tissue was obtained from adult healthy European Shorthair cats (n = 10, five males and five females, aged approximately 1 year) and adult healthy Beagle dogs (n = 4, two males and two females, aged from 2 to 3 years) directly after euthanasia and samples were quickly frozen in liquid nitrogen and stored at −70°C. The cats and dogs had served as controls in authorized studies and the animals were sacrificed with permission of the Animal Ethical Committee and according to the Dutch law on Animal Experiments.

RNA was isolated from 30 mg frozen liver tissue by a spin column purification technique (SV Total RNA Isolation System, Promega, Medison, USA). Aliquots of the purified RNA were measured spectrophotometrically and the RNA was stored at −70°C. cDNA was synthesized using the protocol of the SuperScript III Reverse Transcriptase Kit (Invitrogen, California, USA). The reaction mixture, containing 1 μL of 50 μM oligo(dT)-anchor primer or a gene specific primer (Eurogentec S.A., Belgium), 1μg feline RNA or 2 μg canine RNA and 1 μL 10 mM dNTP Mix (Promega, Madison, USA) in a total volume of 13 μL, was incubated for 5 min at 65°C. Hereafter, the mixture was incubated on ice for at least 1 min and 4 μL of 5× First-Strand buffer (250 mM Tris–HCl pH 8.3, 375 mM KCl, 15 mM MgCl_2_), 1 μL of 0.1 M Dithiothreitol (DTT) and 400 U of SuperScript III Reverse Transcriptase were added to a total of 20 μL reaction volume. This final mixture was incubated for 60 min at 50°C and was inactivated by incubating it for 15 min at 70°C. The cDNA was stored at 4°C until use.

For quantitative Polymerase Chain Reaction (PCR), cDNA was synthesized using the protocol of iScript cDNA Systhesis Kit (Bio-Rad, CA, USA), using 1 μg of feline or canine RNA.

### Sequence analysis

The sequence of Bsep cDNA from the canine and feline liver samples was analyzed using canine Bsep (Abcb11) specific primers, which were based on highly conserved regions between human and canine BSEP/Bsep [The National Centre for Biotechnology Information (NCBI) accession numbers for human: NM_003742; for dog: NM_001143932]. The primer sequences are given in Table 1 and were produced by Eurogentec S.A. Belgium. PCR was performed in a reaction mixture containing a final concentration of 1× Phusion Master Mix (2× Phusion Master Mix contained 0.04 U/μL Phusion DNA Polymerase, 2× Phusion HF Buffer with 3.0 mM MgCl_2_ and 400 μM of each dNTP) (Finnzymes, Espoo, Finland), 1 μM forward primer, 1 μM reverse primer and 1 μL template cDNA in a total of 20 μL. The 3′-end and the 5′-end were obtained by means of a 3′RACE-PCR and 5′-RACE-PCR respectively, using a PCR-anchor primer. After an initial denaturation at 98°C for 30 s, PCR was performed at 98°C for 10 s, at 50-60°C for 30 s, and at 72°C for 30 s for a total of 35 cycles, followed by a final extension of 7 min at 72°C. The PCR-products were stored at 4°C until for further analysis.

**Table 1:** Designed primers used for PCR and DNA sequencing (Eurogentec S.A., Belgium)

**Primer**	**Sequence (5′→3′)**	**Position (relative to human BSEP [NM_003742])**
Oligo(dT)-anchor	5′-GACCACGCGTATCGATGTCGACTTTTTTTTTTTTTTTT	
PCR-anchor	5′-GACCAGGCGTATCGATGTCGAC	
BSEP_RACE_A	5′-AGTGTTGTTTGCCTGTAGC	3615-3633
BSEP_RACE_B	5′-TGAAGAAAATCGAAACAAT	273-291
BSEP_RACE_C	5′-TGTGCCAAAAATGAGGAG	367-381
BSEP_RACE_D	5′-AGTTCTTGTAATTCAGTGTCA	405-425
BSEP_Af	5′-CTCGACCTGATACGCAAGTTCTGA	3392-3415
BSEP_Ar	5′-AATGGCCCGAGCAATAGCAATAC	3805-3825
BSEP_Bf	5′-CAAGGGAAGGTGATGATAGATGG	3526-3548
BSEP_Br	5′-GATGGGGGCTCCTGTGGTAACT	4065-4086
BSEP_Cf	5′-TGTGCTTCTTCCCCTTCTTGGCT	2840-2862
BSEP_Cr	5′-TGCCCATCTATCATCACCTTCC	3530-3548
BSEP_Df	5′-CAACGCTCCAAGTCTCA	2215-2231
BSEP_Dr	5′-GTGCGGATATTACTGAG	2956-2972
BSEP_Ef	5′-CAAGGCTTGCTACGGATGC	2702-2720
BSEP_Er	5′-TGATTGGGGGTTGTCGATC	3295-3310
BSEP_Ff	5′-GTGGTGGCCAGAAACAAAG	1805-1823
BSEP_Fr	5′-TCTGGCTGAATAAAAAGGCA	2442-2461
BSEP_Gf	5′-TGGATCGAATTAAGGGTGAA	1364-1383
BSEP_Gr	5′-GCGATGAGCAACCGAAATGA	1955-1974
BSEP_Hf	5′-GATGGGATTCTTTACTGGATTC	1089-1110
BSEP_Hr	5′-CCTTCACTGGGGTCATAGAA	1537-1556
BSEP_If	5′-GCAGCTCGTCAGATACAGAA	625-644
BSEP_Ir	5′-CAGAAGGCCAATGCATAACA	1132-1151
BSEP_Jf	5′-TGAAGGCCTATGCCAAAGC	938-956
BSEP_Jr	5′-TTCACCCTTAATTCGATCCA	1364-1383
BSEP_Kf	5′-ATTGTTTCGATTTTCTTCA	273-291
BSEP_Kr	5′-GCTTTGGCATAGGCCTTCA	938-956
BSEP_Lf	5′-AACCCTTGTCCAGATTTTCCTC	1197-1218
BSEP_Lr	5′-ATGATGGGTTTCCGGTCTATTG	1313-1334
FullBsep_felinef	5′-TTGCAATTACCATGTCTGACTCAGTAATTCTTCGC	
FullBsep_feliner	5′-TCAACTGATGGGGGCTCCTGTGATGACTAG	
FullBsep_caninef	5′-TTGCAATTACCATGTCTGATGCAGTAATTCTTCGC	
FullBsep_caniner	5′-TCAACTGATGGGGGCTCCTGTGGTAACTAG	
FullBSEP + AttB1_humanf	5′-GGGGACAAGTTTGTACAAAAAAGCAGGCTTCGCCA CCATGTCTGACTCAGTAATTCTTC	
FullBSEP + AttB1_humanr	5′-GGGGACCACTTTGTACAAGAAAGCTGGGTCTCAAC TGATGGGGGATCCAGTG	
AttB1_felinef	5′-GGGGACAAGTTTGTACAAAAAAGCAGGCTTCGCC ACCTTGCAATTACCATGTCTGACTCAGTAATTCTTCGC	
AttB1_feliner	5′-GGGGACCACTTTGTACAAGAAAGCTGGGTCTCAAC TGATGGGGGCTCCTGTGATGACTAG	
AttB1_caninef	5′-GGGGACAAGTTTGTACAAAAAAGCAGGCTTCGCC ACCTTGCAATTACCATGTCTGATGCAGTAATTCTTCGC	
AttB1_caniner	5′-GGGGACCACTTTGTACAAGAAAGCTGGGTCTCAAC TGATGGGGGCTCCTGTGGTAACTAG	

The Oligo(dT)-anchor, PCR-anchor and the specific primer BSEP_RACE_A, were used for 3′RACE-PCR. The BSEP_Ar, Oligo(dT)-anchor, PCR-anchor, BSEP_RACE_B, C and D were used for 5′RACE-PCR. Each primer pair is denoted with “f” for the forward primer, and “r” for the reverse primer.

The PCR products were separated by gel electrophoresis (1.2% agarose gel stained with ethidium bromide (Bio-Rad, CA, USA)) and the cDNA was extracted and purified from the gel by a spin column technique (Wizard SV Gel and PCR Clean-Up System kit, Promega, Madison, USA). The samples were further processed with the ABI PRISM BigDye Terminatior v3.1 Ready Reaction Cycle Sequencing Kit (Applied Biosystems, Foster City, USA), purified by Sephadex G-50 Superfine (Amersham Biosciences, NJ, USA) and sequenced by an automated DNA sequencer (ABI PRISM 3130xl, Applied Biosystems). The cDNA sequences of the feline and canine Bsep were assembled and the predicted protein sequence was derived from the open reading frame.

### Hepatic mRNA expression of feline and canine Bsep (Abcb11)

The expression of Bsep cDNA in feline and canine liver samples was evaluated by RT-PCR using SYBR Green Supermix (Bio-Rad, CA, USA) and species-specific primers (Table 1) and gel electrophoresis. After a hot start of 95°C for 3 min, PCR was performed at 95°C for 20 s, at 55-65°C for 30 s, and at 72°C for 30 s for a total of 40 cycles.

### Cloning of cDNA encoding Bsep/BSEP (Abcb11/ABCB11)

To isolate the full-length Bsep encoding sequence, a PCR was performed as described before with primers specific for each animal species. Primers are given in Table 1. For the cat the set of primers consisted of the forward and reverse primers FullBsep_felinef and FullBsep_feliner respectively. For the dog, the forward primer FullBsep_caninef and the reverse primer FullBsep_caniner was used. The product obtained from this first PCR was used for a second PCR, to attach AttB1-primers used for the cloning procedure of Bsep into the membrane vesicles of HEK293 cells (Gateway, Invitrogen, CA, USA). The conditions of the PCR analysis were similar as described above, except for a final concentration of approximately 9% DMSO in the PCR mixture, an annealing temperature of 70°C and a total of 10 cycles. AttB1-primers used for this second PCR are given in Table 1. A PCR reaction to obtain the full-length human BSEP encoding sequence was performed on the ORFEXPRESS™-ABCB11 vector (LabOmics, GC-H5308) with the forward primer FullBSEP + AttB1_humanf and the reverse primer FullBSEP + AttB1_humanr. After obtaining the BSEP-AttB-product of each species, the product was purified with 30% polyethylene glycol (PEG) 8000/30 mM MgCl_2_ to remove the surplus of primers.

The pENTR221-BSEP vectors for all species were constructed by performing a BP-reaction. Toxicity problems due to a TATAAT sequence in the human BSEP gene [12] were circumvented by introducing a silent mutation at base pair position 81 (AAT →AAC). The sequences of the BSEP/Bsep genes within the pENTR221-BSEP vectors were confirmed, and an LR reaction was performed with the pENTR221 vectors containing the feline, canine and human Bsep/BSEP and with the destination vector BacMam-VSV-DEST. Enhanced yellow fluorescent protein (eYFP) was used as a negative control, as described previously [13]. Eventually, the expression vectors BacMam-VSV-EX-BSEP were constructed for all species.

### Preparation of membrane vesicles of HEK293 cells expressing Bsep/BSEP

After production of recombinant baculo-viruses following the Bac-to-Bac manual, HEK293 cells were transfected and membrane vesicles were isolated as previously described [13]. Briefly, transfected HEK293 cells were lysed with a hypotonic buffer and centrifuged at 100.000 *g.* Pellets were resuspended in an isotonic buffer and the membrane vesicles were isolated with spinning steps of 4000 *g* and 100.000 *g*. After passing the membranes through a 27-gauge needle for 25 times, protein concentrations were determined with the Bio-Rad protein assay kit (Bio-Rad, CA, USA). Presence of BSEP protein in the vesicles was demonstrated by Western blotting using a polyclonal antibody against rat Bsep, which was a generous gift from Dr. B. Stieger (University of Zürich, Switzerland). The antibody was produced in rabbits against the last 13 amino acids of rat Bsep (amino acid sequence: AYYKLVITGAPIS) [12] and a secondary goat-anti-rabbit HRP antibody. As a control, Western blotting was first performed on liver tissue of rats, cats and dogs. Protein concentrations were determined according to Bradford [14].

### Functional characterization of Bsep/BSEP-containing membrane vesicles

The membrane vesicles (7.5 μg protein) were incubated in a 30 μL transport mixture with a final concentration of 10 mM Tris Base (pH 7.4), 250 mM sucrose, 10 mM MgCl_2_, 4 mM adenosine triphosphate (ATP) or adenosine monophosphate (AMP), 1 μM [^3^H]taurocholic acid (TCA), with or without an inhibitor, in 96-well plates at 37°C. For the concentration-dependent curves 0.15 μCi [^3^H]TCA, 0.05 μCi [^3^H]GCA and 0.3μCi [^3^H]CA was used, supplemented with unlabeled TCA, GCA or CA respectively. The reaction was stopped by placing the 96-well plate on ice-water and by adding 150 μL ice-cold washing buffer (10 mM Tris Base pH 7.4, 250 mM sucrose). The reaction mixture was transferred to a 96-well Multi-Screen HTS filter plate (Millipore, Ireland) and the total mixture was filtered by means of a Multi-Screen HTS vacuum manifold filtration device (Millipore, Etten-Leur, The Netherlands). The filters were washed twice with 200 μL washing buffer, whereafter they were separated from the plate. Two ml scintillation fluid was added and the radioactivity remaining on the filter was measured by a liquid scintillation analyzer (Tri-carb 2900 TR, Packard). ATP-dependent transport was calculated by subtracting uptake in presence of AMP from that in presence of ATP. The incubation period was checked for linearity in transport rate. Concentration-dependent BSEP transport rates were fitted according to Michaelis-Menten enzyme kinetics and K_m_ values were calculated by means of GraphPad Prism 6.01 software (San Diego, California, USA).

### Statistical analysis

Data were expressed as means ± SD of at least three independent experiments with samples performed in duplicate. Data were analyzed using a one-way or two-way analysis of variance (ANOVA) followed by the Bonferroni post-hoc test (GraphPad Prism 6.01 software, San Diego, California, USA) with P < 0.05 denoting a significant difference.

## Results

### cDNA sequence and predicted amino acid sequence of feline and canine Bsep

The cDNA coding sequences of feline and canine Bsep were obtained as described in the Methods section and demonstrated a high level of homology with an identity of 91.2%. The feline cDNA sequence has been submitted to the NCBI database [NCBI accession number KF601333]. The open reading frame (ORF) of the obtained canine Bsep sequence was highly identical (99.8%) to the previously reported sequence by Yabuuchi et al. [11], with only a few non-coding differences in the cDNA sequence resulting in a completely similar amino acid sequence. Feline Bsep cDNA was 89.3% identical to human BSEP sequence [NCBI accession number NM_003742]. In this respect, dogs also share 89.3% identity with human BSEP.

**Figure 1:**
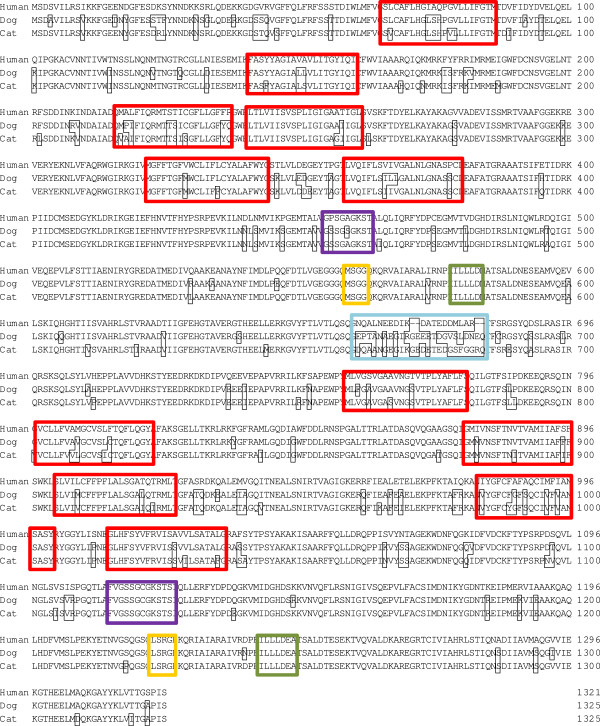
**Alignment of human, dog and cat amino acid sequences of BSEP/Bsep ORF.** Differences in amino acid pattern relative to the human BSEP sequence are given in black borders. Transmembrane domains were predicted by Yabuuchi et al. [11] for the dog, and are given in red borders for all species. Walker A is given in purple borders, Walker B in green borders and Signature C in yellow borders. The region of highly different amino acid sequences is given in a blue border.

The amino acid sequence deduced from the ORF of ABCB11 of the different species is shown in Figure 1. Differences in amino acids between human, dogs and cats are displayed in small black borders. The amino acid sequences showed a similarity of 92.3% between cats and dogs. Cat Bsep is 88.9% identical to the human protein, and dog Bsep 89.6%. The twelve transmembrane domains, the Walker A and B motifs and the signatures were highly identical between species. The major differences in amino acid pattern were located halfway the ORF sequence (ORF position 661–684), in the “linker domain” of the first nucleotide-binding domain, over a range of 23 amino acids. Moreover, both feline and canine ORF sequences contained four amino acids more than the human BSEP ORF in this region.

### Bsep/BSEP gene and protein expression in liver tissue samples

**Figure 2:**
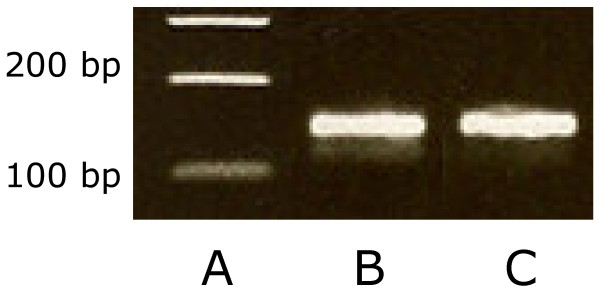
**Bsep product of canine and feline liver tissue using primers BSEP_Lf and BSEP_Lr on a 1.8% agarose gel stained with ethidium bromide. A**: BenchTop 100 bp DNA Ladder (Promega, USA); **B**: canine Bsep; **C**: feline Bsep.

Presence of *Bsep* mRNA in pooled liver samples from cats and dogs was demonstrated by PCR analyses (Figure 2). The bands of *Bsep* mRNA can only be compared qualitatively, but they appeared to be of similar density. Presence of Bsep protein in liver tissue was demonstrated by Western blotting using a rabbit anti-rat Bsep antibody. The expression of Bsep was observed in livers from rat, dog and cat (data not shown).

### Bsep/BSEP protein expression in membrane vesicles prepared from native and Bsep/BSEP-overexpressing HEK293 cells

**Figure 3:**
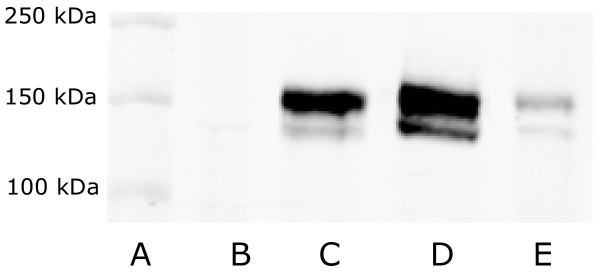
**Western Blot of membrane vesicles over-expressing cat, dog and human Bsep/BSEP. A**: Precision Plus Protein Dual Color Marker (Bio-rad Laboratories); **B**: eYFP (control); **C**: Bsep cat; **D**: Bsep dog; **E**: BSEP human. In lanes **B**, **C**, **D**, and **E** equal amounts of total protein were loaded.

The presence of Bsep/BSEP in membrane vesicles prepared from HEK293 cells overexpressing feline, canine and human Bsep/BSEP was confirmed by Western blotting (Figure 3). No staining was seen in the vesicles serving as negative control that were prepared from mock-transduced HEK293 cells with eYFP, which indicates that both bands in the Western blot (lane C, D, and E) are specific. The double band can be explained by posttranslational modifications (most likely N-glycosylation) of the protein as previously discussed by Gerloff et al. [15].

### Vesicular uptake studies

Vesicular uptake of tritium-labeled TCA, GCA and CA was demonstrated for all samples containing Bsep/BSEP proteins, while uptake into membrane vesicles prepared from mock-transduced HEK293 cells did not exceed background activity (data not shown). The vesicular uptake was ATP-dependent and the rate of uptake was time-dependent (data not shown). To be in the linear range of the uptake rate, samples were incubated for 5 minutes to assess the transport kinetics of TCA and 7.5 minutes to assess the transport kinetics of GCA and CA.

**Figure 4:**
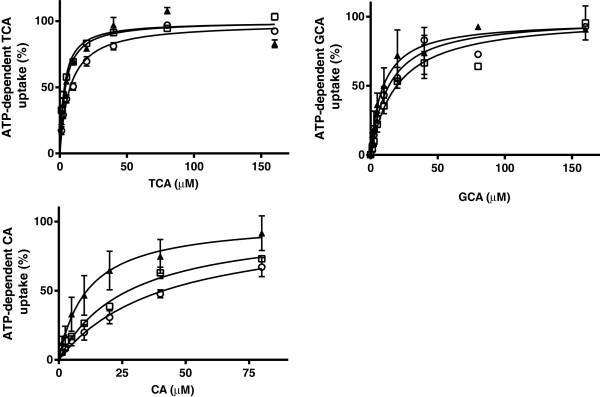
**ATP-dependent transport of TCA, CA and GCA in membrane vesicles (7.5 μg protein) expressing BSEP/Bsep of human (▲), dogs (□), and cats (○).** Vesicles were incubated in 10 mMTris Base buffer (pH 7.4) containing 250 mM Sucrose, 10 mM MgCl_2_ and 4 mM ATP or AMP, at 37°C for 5 min (TCA) or 7.5 min (CA and GCA). ATP-dependent transport was calculated by subtracting transport in presence of AMP from that in presence of ATP. Curves were fitted by GraphPad Prism 6.01 software (San Diego, California, USA) and V_max_ was calculated. Measurements were performed in duplicate in at least three independent experiments. The mean of the duplicates of each experiment was transformed as a relative activity to the V_max_ (=100%). Each value in the graph is the mean ± SD of the relative activities of three independent experiments.

**Figure 5:**
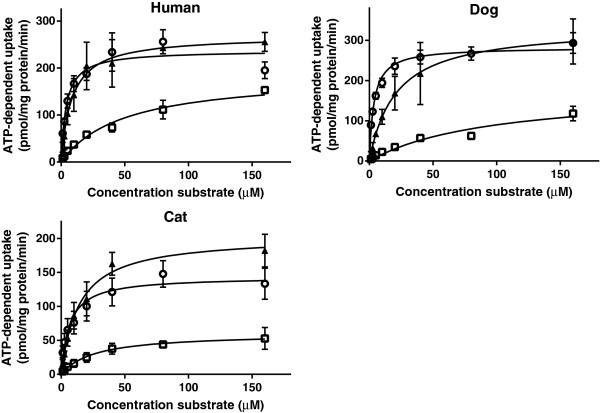
**ATP-dependent transport of TCA (○), GCA (▲) and CA (□) in membrane vesicles (7.5 μg protein) expressing human, dog and cat BSEP/Bsep.** Vesicles were incubated in 10 mMTris buffer (pH 7.4) containing 250 mM Sucrose, 10 mM MgCl_2_ and 4 mM ATP or AMP, at 37°C for 5 min (TCA) or 7.5 min (CA and GCA). ATP-dependent transport was calculated by subtracting transport in presence of AMP from that in presence of ATP. Curves were fitted by using GraphPad Prism 6.01 software (San Diego, California, USA). Measurements were performed in duplicate in three independent experiments and are given in pmol/mg protein/min. Each value in the graph is the mean ± SD of three independent experiments.

The rate of bile salt uptake into the vesicles was saturable and the data could be described according to Michaelis-Menten kinetics (Figures 4 and 5). K_m_ values obtained for each bile acid from nonlinear regression analysis were compared between species (Table 2). There were no differences in K_m_ values of each bile acid between the vesicles prepared from cells overexpressing Bsep/BSEP of the different species. Within species, the K_m_ value for uptake of CA by the vesicles overexpressing feline or canine Bsep was higher than the corresponding K_m_ value for TCA uptake. The feline Bsep also had a lower affinity for CA than for GCA.

**Table 2:** **K**_**m **_**values for TCA, GCA and CA of human, dog and cat BSEP/Bsep containing membrane vesicles (7.5 μg protein)**

	**Km (μM)**		
	**Human**	**Dog**	**Cat**
TCA	4.1 ± 0.5	3.4 ± 0.3	7.5 ± 0.7
GCA	9.0 ± 2.0	19.3 ± 3.5	13.4 ± 2.0
CA	11.3 ± 4.3	27.2 ± 3.2^a^	41.2 ± 11.3^b,c^

^a^significantly different from TCA in dogs.

^b^significantly different from TCA in cats.

^c^significantly different from GCA in cats.

K_m_/V_max_ curves were fitted by GraphPad Prism 6.01 software (San Diego, California, USA) and K_m_ was calculated. Measurements were performed in duplicate in at least three independent experiments and mean K_m_ ± SD are given in the table. Statistical significance was determined by a two-way ANOVA followed by the Bonferroni post-hoc test. P < 0.05 indicates a significant difference.

Differences in maximum rate of ATP-dependent uptake were seen between uptake of CA versus TCA and GCA. Variations in the maximum rate of ATP-dependent uptake were also seen in the uptake of the bile acids between the species (Figure 5).

**Figure 6:**
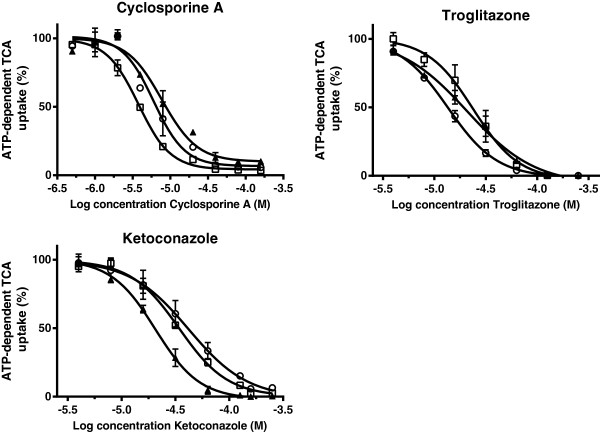
**Inhibition of cyclosporine A, troglitazone and ketoconazole on the uptake of 1 μM [**^**3**^**H]TCA in membrane vesicles (7.5 μg protein) expressing human (▲), dog (□) and cat (○) BSEP/Bsep.** Vesicles were incubated in 10 mMTris Base buffer (pH 7.4) containing 250 mM Sucrose, 10 mM MgCl_2_ and 4 mM ATP or AMP, at 37°C for 5 min. ATP-dependent transport was calculated by subtracting transport in presence of AMP from that in presence of ATP. Inhibition curves were fitted by using GraphPad Prism 6.01 software (San Diego, California, USA) and values are expressed as mean ± SD of percentage uptake of fourindependent experiments.

The inhibitory effects of three model Bsep inhibitors were subsequently determined on TCA uptake in the membrane vesicles. Cyclosporine A, troglitazone and ketoconazole decreased the uptake of TCA by vesicles prepared from the cells overexpressing Bsep/BSEP of the different species and the data were fitted by nonlinear regression to a one-site competition model (Figure 6). Inhibitory potencies, as given by the IC50 values, were all in the micromolar range and the potency of each compound did not differ significantly between species (Table 3).

**Table 3:** **Inhibitory potency of cyclosporine A, troglitazone and ketoconazole against 1 μM [**^**3**^**H]TCA uptake into membrane vesicles (7.5 μg protein) expressing human, dog and cat BSEP/Bsep**

	**IC**_**50 **_**(μM)**		
	**Human**	**Dog**	**Cat**
Cyclosporine A	7.8 ± 1.2	3.8 ± 0.2	6.0 ± 0.8
Troglitazone	20.9 ± 2.9	23.9 ± 2.5	13.6 ± 4.2
Ketoconazole	20.6 ± 1.1	34.2 ± 2.0	41.8 ± 6.6

Inhibition curves were fitted by using GraphPad Prism 6.01 software (samples without inhibitor were set on 100%). [^3^H]TCA uptake in presence of ATP was corrected for uptake in presence of AMP. Measurements were performed in duplicate in four independent experiments. Statistical significance was determined by one-way ANOVA followed by the Bonferroni post-hoc test with P < 0.05 denoting a significant difference. No significant differences were found between the different species.

## Discussion

The aim of this study was to characterize and compare the function of feline bile acid efflux transporter Bsep with canine and human Bsep/BSEP with respect to endogenous bile acids and typical drug inhibitors. To this end, membrane vesicles isolated from genetically engineered cells overexpressing Bsep/BSEP of the different species were made. To our knowledge, this is the first study in which feline Bsep has been cloned and characterized.

The cDNA sequence of the ORFs of feline, canine and human Bsep/BSEP showed a high similarity between the species. Analysis of the translated protein sequences revealed that the twelve transmembrane domains, which are thought to be responsible for substrate specificity, and the Walker A and Walker B motifs, which are needed for binding and hydrolyzation of ATP, appeared to be highly identical. A more divergent part of the protein was observed in the linker region from amino acid 661 to 684, where feline and canine Bsep contains four additional amino acids compared to the human BSEP. The linker region may have a regulatory role in the rate of ATP hydrolysis, as has been described for P-glycoprotein/ABCB1 [16], or it could mediate the turnover of the ABC transporter [17]. Amino acid differences in the linker region could also relate to species differences in post-transcriptional regulation of BSEP/Bsep.

The presence of BSEP/Bsep protein in the membrane vesicles that were prepared from HEK293 cells overexpressing the transporter was confirmed by Western blotting. Although differences in BSEP/Bsep staining intensity were observed between vesicle preparations of the different species, a quantitative estimate of transport protein abundance could not be made. The used antibody was developed against the last 13 amino acids of the ORF of rat Bsep and differences in affinity for the proteins of the different species could be expected. The terminal 13 amino acids of rat Bsep are exactly the same for feline Bsep. Canine Bsep differs one amino acid and human BSEP two amino acids from rat. Variations in transporter abundance in the vesicle preparations may have occurred and a direct comparison of maximum transport activities of BSEP/Bsep between the different species can therefore not be made. However, we observed in different transductions and vesicle preparations consistently lower Vmax values of cat Bsep for all tested bile acids compared to human and dog BSEP/Bsep. Moreover, in spite of a low staining intensity for human BSEP compared to canine and feline Bsep, we consistently found the highest Vmax for human BSEP for all substrates compared to the other species.

The functional studies demonstrated that TCA, GCA and CA were substrates of BSEP/Bsep in all three species, showing saturable transport kinetics. K_m_ values were in the same range as reported by different groups for human BSEP and rat Bsep in different expression systems [6]. In all species there was a tendency to a higher affinity of Bsep for TCA than GCA, and a higher affinity for GCA than CA. The same order of affinity of the bile acids TCA and GCA was previously noted for human BSEP [12,18]. The lower affinity and maximum transport rate of CA compared to TCA and GCA for feline and canine Bsep, is in accordance with a relatively lower amount of unconjugated bile salts in bile of these species. The conjugated bile acid TCA is a model substrate for BSEP, commonly used in *in vitro* functional studies, although in man bile acids are mainly conjugated to glycine and only to a minor extent to taurine. This is in contrast to carnivore species, like cat and dog, in which bile acids are almost exclusively conjugated to taurine with TCA as the major bile acid in both species [19]–[21]. Since protein sequence and function of Bsep appeared to be highly conserved among human, dog and cat, inter-species variation in the constitution of conjugated bile acids must be more related to the availability of co-substrates or enzymes for conjugation.

The inhibitory potency of the model inhibitors cyclosporine A, troglitazone and ketoconazole was characterized against TCA uptake into BSEP/Bsep containing membrane vesicles. All three substances potently inhibited TCA uptake without significant species differences. A comparable inhibitory potential of troglitazone was found previously for human and canine BSEP/Bsep [11,18].

## Conclusions

The structure and functional characteristics of cat Bsep appeared to be very similar to dog and human Bsep/BSEP, indicating that the properties of this transporter have been highly preserved among the different species. The methods and results of this study can be used as an *in vitro* model for the assessment of interactions of drugs and other substances with feline and canine Bsep, which is suitable to study the risk of drug-induced cholestasis in these species in more detail.

Inhibition of BSEP by drugs has been related to cholestasis and subsequent drug-induced liver injury in man and could also be a mechanism in cholestasis and liver disease in veterinary relevant animal species.

## Competing interests

The authors declare that they have no competing interests.

## Author’s contributions

CB and JH carried out all the experimental work and CB drafted the manuscript. JK, JS and FR provided valuable information on the subject of ABC-transporters, the design of the study and revised the manuscript. JS and FR coordinated and supervised the study. All authors read and approved the final manuscript.
